# Is Anxiety Best Conceived as a Unitary Condition? The Benefits of Lumping Compared With Splitting . . .

**DOI:** 10.1177/070674371405900601

**Published:** 2014-06

**Authors:** Margaret A Richter

**Affiliations:** 1 Head of Frederick W Thompson Anxiety Disorders Centre, Sunnybrook Health Sciences Centre, Toronto, Ontario; Associate Professor of Psychiatry, University of Toronto, Toronto, Ontario.

**Keywords:** anxiety disorders, nosology, psychiatric disorders, Diagnostic and Statistical Manual of Mental Disorders—Fifth Edition

There has been a growing tendency in psychiatric nosology toward increasing specificity of diagnostic syndromes, reflecting an underlying common belief that division of conditions into more homogenous groups will assist in our efforts to better determine the underlying neurobiology, specific conceptualization, and thereby treatment course of these differing disorders. This has been exemplified in the changes over the years leading to the DSM-5.^1^ The number of discrete psychiatric diagnoses have grown substantially, from 106 in the DSM-I to more than 250 in the DSM-IV-TR and DSM-5. As a result of this increase in the number of recognized psychiatric disorders, more than 46% of the US (and presumably Canadian) population will meet criteria for one or more psychiatric diagnosis during their lifetime.^2^ This rather simple observation underscores that inflation in identification of people categorized as unwell is a potential unfortunate byproduct of this process. An alternative classification for research purposes are the RDoCs, which emphasize dimensions and quantification of symptoms, rather than categories. The RDoCs are proposed to be better equipped to address the underlying neurobiology, given that neither DNA acid nor neurocircuits read the DSM.^3^

The coexistence of RDoCS and the DSM-5 reflects the tension between those who would prefer to take a broader perspective of illness and those who support efforts to divide illness into evermore precise and narrowly defined categories. The term lumping and splitting has been used to describe these warring tendencies. First used by Charles Darwin, this term has been applied to disease and other classification systems, such as “On Lumpers and Splitters, or the Nosology of Genetic Disease”,^4^ a 1969 paper by Victor A McKusick, who is viewed as the father of contemporary clinical medical genetics. This example is particularly apt, as one major argument for refinement of psychiatric nosology has been the hope that it will advance understanding of the genetic roots of psychiatric disorders. Interestingly, although identification of genetic risk factors has been an area in which increasing specificity of the phenotype has been widely viewed as a necessity, recent landmark work from the Cross-Disorder Group of the Psychiatric Genomics Consortium^5^ highlights the benefit of this cross-diagnostic boundary approach. Examination of pooled genome-wide genotype data for schizophrenia, BD, MDD, autism spectrum disorder, and attention-deficit hyperactivity disorder identified high genetic correlation between schizophrenia and BD (*r* = 0.68, SE = 0.04), and moderate correlation between illnesses as phenomenologically diverse as schizophrenia and MDD (*r* = 0.43, SE = 0.06) as a result of shared common single-nucleotide polymorphisms. Studies such as this appear to fly in the face of the long-accepted wisdom of drilling down to define increasingly homogeneous subgroups.

Similarly, understanding of the neurobiological underpinnings of psychiatric disorders may also be moving toward identification of more commonality than was previously thought. A good example of this is the increasing recognition of the role of glutamate neurotransmission in multiple disorders. There is evidence of its vital role in schizophrenia,^6^ depression,^7^ and OCD.^8^ Further, glutamatergic mechanisms are seen as increasingly important in the other anxiety disorders via their importance in learning, neuroplasticity, and fear extinction, leading to exploration of glutamate receptors as novel targets for pharmacotherapy of these disorders.^9^,^10^

Regarding the anxiety disorders, it certainly appears that splitting has been the reigning paradigm, to date. Conditions formerly grouped together as the anxiety disorders in DSM-IV are now divided into 3 DSM-5 chapters, with PTSD and acute stress disorder included with trauma- and stressor- related conditions, and (OCD) listed in a new obsessive–compulsive and related disorders chapter. This separation is intended to reflect differences in the underlying neurocircuitry of OCD, compared with the other anxiety disorders and corresponding characteristic distinctions in treatment for this condition.^11^ However the meta-structure of the DSM-5 also reflects the presumed relatedness of these conditions by making these chapters adjacent to one another.^12^

The 2 papers in this issue of *The Canadian Journal of Psychiatry* are of particular interest as they essentially buck this trend toward ever-increasing specificity and explore the clinical utility of instead lumping the anxiety disorders together, from very different perspectives. The paper by Mr Andrew Peterson and colleagues^13^ reviews recent functional neuroimaging research of resting-state connectivity between brain regions in the various anxiety disorders, including OCD and PTSD. As the authors point out, this literature has been hampered by the use of markedly different paradigms in individual disorders, making any exploration of differences or commonalities across these groups very challenging. The authors thus chose to examine activity in intrinsic cognitive networks in the resting state, and in the process identified some overlap in the neural networks underlying the different disorders. This work thus highlights broad similarities across disorders, such as altered connectivity between limbic regions, including bilateral amygdale, insula, and amygdalar subregions in PTSD, generalized anxiety disorder, social anxiety disorder, and panic disorder, albeit, in variable ways. Concurrently, this review highlights differences unique to specific disorders, such as involvement of the corticostriatal networks in OCD.

The review by Dr Neil A Rector and colleagues^14^ explores this issue by examining the development of transdiagnostic CBT for the anxiety disorders.^14^ These treatments focus on higher-order factors shared across the different anxiety disorders, along with MDD, as it is highly comorbid with these conditions, such as increased negative affectivity. Notwithstanding the excellent efficacy of targeted disorder-specific treatment protocols, the high rate of comorbidity between these disorders poses a significant problem for the clinician in determining what to target first. These transdiagnostic approaches, such as the Unified Protocol, have been designed to be used in either straightforward or complex comorbid cases, with modules focusing on skills, such as cognitive flexibility and exposure and (or) reduction of situational avoidance. While the transdiagnostic approaches appear to be producing moderate-to-large effects (*d* = 0.5 to 1.4), and a significant per cent of participants no longer meet criteria for any diagnosis by the end of a transdiagnostic protocol, no study has met or exceeded the very large treatment effects commonly seen in disorder-specific CBT.

Taken together, this literature, of both the basic neuroscience and psychological treatment of these conditions, appears to provide surprising support for the potential advantages of a broader perspective on the seemingly disparate anxiety-related disorders, and suggest some surprising benefit of the lumping paradigm. Neurobiological research may benefit from use of broader-based or dimensional disease models. Similarly, the potential benefits of the newer transdiagnostic treatments warrant closer inspection. Currently, it is unclear how well these approaches compare to evidence-based standard CBT treatments. As discussed by Dr Rector and colleagues,^14^ the individual novel elements being introduced need further study in well-designed protocols to best optimize this kind of treatment. However, this development does hold the important promise of brief, readily mastered protocols that will potentially prove more efficacious for the numerous people presenting with more than one disorder warranting clinical treatment. Concurrently, this work points to the need for researchers working in this area to be able to step back and approach our investigative questions with greater cognitive flexibility ourselves, considering whether the questions we are asking are best served by exploring broader anxiety constructs or more narrowly-defined disorders. Like most issues in research, it is unlikely that one approach will be best for all.

## Abbreviations

BD: bipolar disorder
CBT: cognitive-behavioural therapy
DSM: Diagnostic and Statistical Manual of Mental Disorders
MDD: major depressive disorder
OCD: obsessive–compulsive disorder
PTSD: posttraumatic stress disorder
RDoCs: Research Domain Criteria
